# A patient advocating for transparent science in rare disease research

**DOI:** 10.1186/s13023-022-02557-6

**Published:** 2023-01-19

**Authors:** Richard Rui Yang

**Affiliations:** Reflection Biotechnologies Limited, Unit 601, 6/F, Core Building 1, No. 1 Science Park East Avenue, Pak Shek Kok, New Territories, Hong Kong China

**Keywords:** Patient perspective, Transparent science, Rare Disease Research, Orphan Drug Development, Cyp4v3 knockout mouse, Bietti Crystalline Dystrophy, Genetic background, Interspecies differences, Animal model flaws

## Abstract

300 million people live with at least one of 6,000 rare diseases worldwide. However, rare disease research is not always reviewed with scrutiny, making it susceptible to what the author refers to as nontransparent science. Nontransparent science can obscure animal model flaws, misguide medicine regulators and drug developers, delay or frustrate orphan drug development, or waste limited resources for rare disease research. Flawed animal models not only lack pharmacologic relevance, but also give rise to issue of clinical translatability. Sadly, these consequences and risks are grossly overlooked. Nontransparency in science can take many forms, such as premature publication of animal models without clinically significant data, not providing corrections when flaws to the model are discovered, lack of warning of critical study limitations, missing critical control data, questionable data quality, surprising results without a sound explanation, failure to rule out potential factors which may affect study conclusions, lack of sufficient detail for others to replicate the study, dubious authorship and study accountability. Science has no boarders, neither does nontransparent science. Nontransparent science can happen irrespective of the researcher’s senority, institutional affiliation or country. As a patient-turned researcher suffering from Bietti crystalline dystrophy (BCD), I use BCD as an example to analyze various forms of nontransparent science in rare disease research. This article analyzes three papers published by different research groups on *Cyp4v3*^−/−^, high-fat diet (HFD)-*Cyp4v3*^−/−^, and Exon1-*Cyp4v3*^−/−^ mouse models of BCD. As the discussion probes various forms of nontransparent science, the flaws of these knockout mouse models are uncovered. These mouse models do not mimic BCD in humans nor do they address the lack of Cyp4v3 (murine ortholog of human CYP4V2) expression in wild type (WT) mouse retina which is markedly different from CYP4V2 expression in human retina. Further, this article discusses the impact of nontransparent science on drug development which can lead to significant delays ultimately affecting the patients. Lessons from BCD research can be helpful to all those suffering from rare diseases. As a patient, I call for transparent science in rare disease research.

## Background

There are more than 6,000 rare diseases, many of which are devastating disorders that causes premature death or life-long disabilities [1]. More than 90% of rare diseases still do not have any effective treatment [2]. Today, many countries and regions in the world have legislations or policies concerning rare diseases and orphan drugs [3, 4]. Policy incentives, increased public funding, and private sector interest have sparked more research on rare diseases. With urgent unmet medical needs, patients rely on researchers from private and public sectors to discover treatments for diseases.

As compared to research on common diseases, rare disease research is not always reviewed with scrutiny, leaving room for what I refer to as nontransparent science, which can obscure animal model flaws, misguide medicine regulators and drug developers, delay or frustrate orphan drug development, or waste limited resources for rare disease research. Flawed animal models not only lack pharmacologic relevance, but also give rise to issue of clinical translatability. Sadly, these consequences and risks are grossly overlooked. Nontransparency in science can take many forms, such as premature publication of animal models without clinically significant data, not providing corrections when flaws to the model are discovered, lack of warning of critical study limitations, missing critical control data, questionable data quality, surprising results without a sound explanation, failure to rule out potential factors which may affect study conclusions, lack of sufficient detail for others to replicate the study, and dubious authorship and study accountability.

As a patient-turned researcher suffering from Bietti crystalline dystrophy (BCD), I use BCD as an example to analyze various forms of nontransparent science in rare disease research and discuss how nontransparent science can impact drug development and ultimately the patients. BCD is a rare blinding disease caused by severe retinal degeneration and mutations in the *CYP4V2* gene. For BCD, there have been three publications that claim to be knockout mouse models of the human disease. The first paper, published in 2014, described the development of a *Cyp4v3* knockout (*Cyp4v3*^−/−^) mouse model of BCD, also referred to in this article as the *Cyp4v3*^−/−^ Mouse Paper [5]. The second paper, published in 2020, pointed out defects of the *Cyp4v3*^−/−^ mouse model and reported that high-fat diet (HFD) induced retinal phenotypes in the *Cyp4v3*^−/−^ mouse, also referred to in this article as the HFD-*Cyp4v3*^−/−^ Mouse Paper [6]. The third paper, published in 2022, pointed out flaws in both the *Cyp4v3*^−/−^ mouse model and the HFD-*Cyp4v3*^−/−^ mouse model, and reported that exon 1 (Exon1)-*Cyp4v3*^−/−^ mice produced surprising phenotypes which recapitulate BCD, also referred to in this article as the Exon1-*Cyp4v3*^−/−^ Mouse Paper [7].

However, nontransparent science obscured flaws in these *Cyp4v3*^−/−^ mouse models and/or may affect credibility of the results or validity of the conclusions of these papers. In addition, these *Cyp4v3*^−/−^ mouse papers did not address the lack of Cyp4v3 (murine ortholog of human CYP4V2) protein expression in wild type mouse retina which is markedly different from CYP4V2 expression in human retina. Besides nontransparent science, limitations to these models will be highlighted in this article to analyze the pharmacological relevance of these *Cyp4v3*^−/−^ mouse models in the development of therapies for BCD.

## Bietti crystalline dystrophy (BCD)

**Fig. 1:**
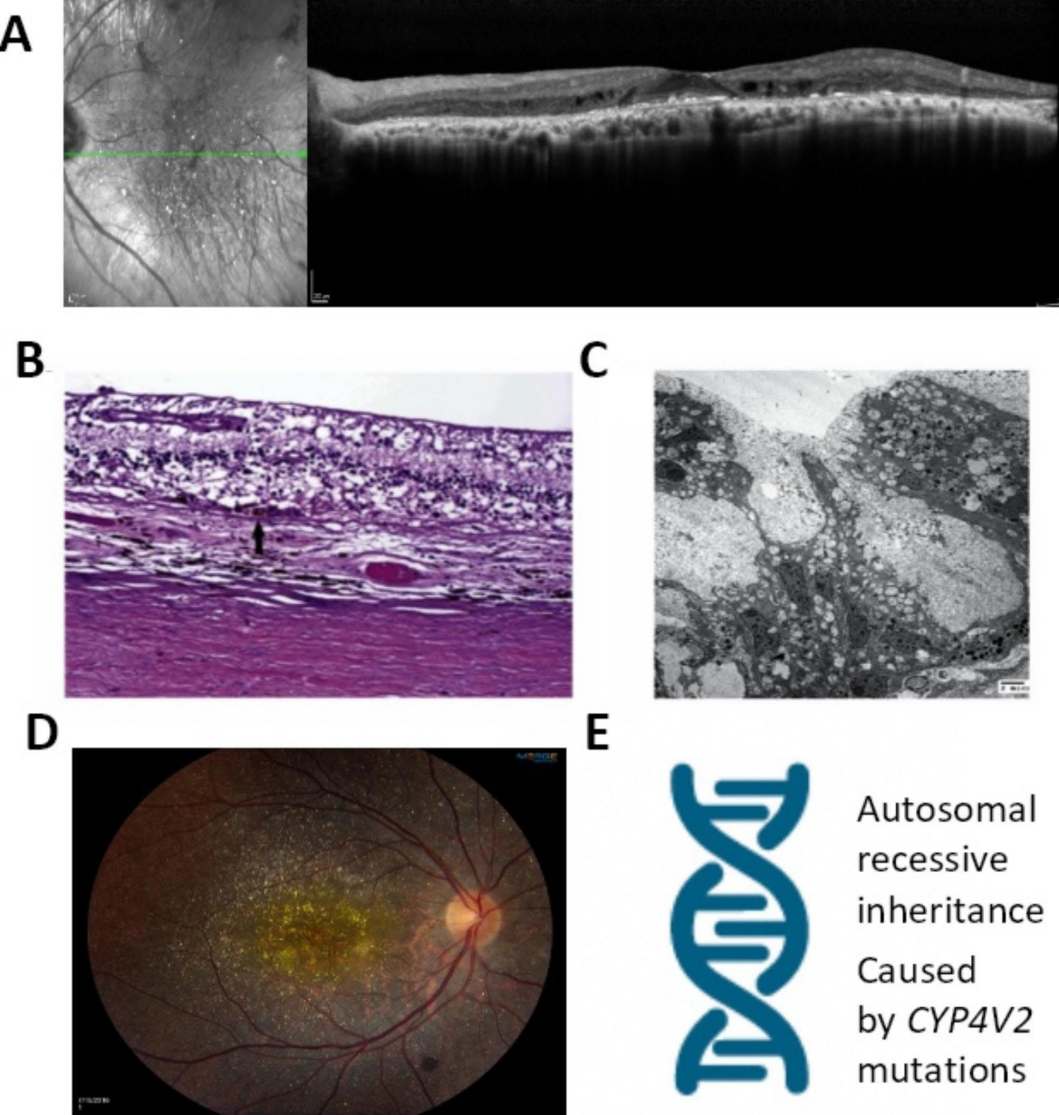
Retinal Phenotype of Bietti Crystalline Dystrophy (BCD). (A) Spectral domain optical coherence tomography (SD-OCT) image of the left eye of a BCD patient (the author). The OCT shows severe RPE and outer retina atrophy and loss of ellipsoid zone (EZ). Hyperreflective subretinal crystals can be seen. (B) Light micrograph of the posterior retina of a BCD patient. There is near total degeneration of all functional elements of the retina leaving only structural astrocytes. Only a small number of recognizable retinal pigment epithelial cells remain (arrow). (hematoxylin-eosin, original magnification × 200). (C) Electron micrograph of the retina of a BCD patient. Retinal degeneration is characterized by intracellular pigmented granules and vacuolated cytoplasm. The retina and choroid also show no evidence of lysosomal crystals. (D) Fundus photograph of the right eye of a BCD patient. There is widespread subretinal crystalline deposits seen throughout the fundus. RPE atrophy is observed at the macula, revealing the underlying choroidal vessels. Some RPE pigment clumps are seen in the peripheral fundus. (E) BCD is an autosomal recessive, retinal degenerative disease caused by mutations in the *CYP4V2* gene. Figure 1B C, and related figure legend descriptions are reproduced from the following open access article [14]: Furusato E, Cameron JD, Chan CC. Evolution of Cellular Inclusions in Bietti’s Crystalline Dystrophy. Ophthalmol Eye Dis. 2010;2010(2):9–15. © 2010 the authors, publisher and licensee Libertas Academica Ltd. Figure 1D was provided with patient consent by Invincible Vision, a BCD patient organization

First described by Italian ophthalmologist G.B. Bietti in 1937 [8], BCD (OMIM 210,370) is a rare, autosomal recessive, progressive, degenerative retinal disease. In 2004, a global study led by the National Eye Institute of the National Institutes of Health (NIH) discovered mutations in the *CYP4V2* gene in patients cause BCD [9]. A devastating blinding disease, BCD is caused by severe retinal degeneration, including severe atrophy of the retinal pigment epithelium (RPE), loss of the outer retina and choriocapillaris (Fig. 1A.) [10–13], with near total degeneration of all functional elements of the retina by the late stage of the disease (Fig. 1B C) [14]. In BCD, abnormal retinal function is evident on diminished electroretinograms (ERG) during the early stage of disease, prior to loss of central vision. This progresses to ERG extinction during the intermediate stage, long before legal blindness occurs [15].

BCD patients have numerous tiny, shiny yellow-white retinal crystals (Fig. 1D), though crystals may disappear in late disease stage [11, 13, 16, 17]. In some patients, crystals have also been found in the cornea [12, 13]. Retinal crystals are not unique to BCD and can be associated with other types of crystalline retinopathies, retinal detachment, drug use, or even high dose of lutein [18–20].

Most BCD patients notice the first symptoms between the second and fourth decade of life, such as night blindness, decreased central visual acuity, or visual field loss. Progressive visual loss and constriction of the visual fields lead to legal blindness usually in the fifth or sixth decade [9, 12, 16]. BCD symptoms are similar to those of other retinal degenerative diseases and is frequently misdiagnosed as Retinitis Pigmentosa (RP), choroideremia, Stargardt disease, Late Onset Retinal Degeneration, or other forms of crystalline retinopathies, or can be completely missed [13, 15, 18, 21]. A prior study reported that only 1 out of 6 BCD patients was initially correctly diagnosed [15], Near-infrared reflectance imaging (NIR) can enhance detection of retinal crystals [10, 22]. Multimodal imaging (fundus photograph, NIR, fundus autofluorescence, and OCT), especially NIR, is useful in BCD differential diagnosis to differentiate BCD patients from patients with other chorioretinal dystrophies accompanied by crystalline-like retinal deposits [23]. Genetic sequencing for *CYP4V2* mutations is the ultimate tool to confirm clinical diagnosis of BCD [9, 10, 12, 13, 17]. Currently, there is no approved treatment available for BCD.

## Animal model flaws and clinical translatability

Traditionally, animal experimentation is viewed as the default and gold standard of preclinical testing in drug development and is generally supported without critical examination of its validity [24]. However, what works in mice or monkeys do not always work in humans. In fact, only about 10% of drugs that work in animal studies are ultimately successful in humans [25]. In some cases, drug candidates based on preclinical animal studies have failed during human clinical trial stage on a massive scale. For example, out of 2,204 clinical trials for Alzheimer’s disease (AD) by July 24, 2017, only four drugs were approved by the U.S. Food and Drug Administration (FDA). [26]. In another case, 150 drugs that successfully treated a sepsis-like condition in mice later failed in human clinical trials—a heartbreaking loss of decades of research and billions of dollars. In fact, this massive failure based on animal model caught the attention of the Director of the NIH [27]. The mouse model failure suggests that we should not assume a mouse’s drug response will always accurately predict a human’s. In addition, it provides more reason to develop better and more sophisticated models of human disease, such as human cell-based models like tissue chips [25, 27]. The use of genetically engineered mice as models has become commonplace in the study of many human genetic diseases. In certain cases, however, knockout mice do not always replicate key clinical phenotypes of human diseases, or have no clinical phenotype at all. Phenotype differences could due to basic differences in mouse and human biology, genetic background or gene interactions, or alternative pathways, which give rise to issues regarding clinical translatability [28–30]. Knock-in and knockout mouse models have also failed to mimic certain retinal degenerative diseases in humans. For example, knockin and knockout mouse models of age-related macular degeneration (AMD) and other allied macular dystro- phies typically lack the same phenotypic manifestations seen in patients [31]. For Choroideremia (CHM), an X-linked retinal disorder which results from defects in the human Rab escort protein-1 (REP-1) gene, disruption of the mouse rep-1 gene gives rise to lethality in male mouse embryos [32]. In addition, there are structural differences between human and mouse retinas [33].

The use of nonpredictive animal experiments can cause human suffering in two ways: by producing misleading efficacy and safety data (false positive prediction), and by causing potential abandonment of useful medical treatments (false negative) and misdirecting resources away from more effective testing methods.[24].

## BCD mouse models

This section reviews three mouse models of BCD, the *Cyp4v3*^−/−^ mouse, the HFD-*Cyp4v3*^−/−^ mouse, and the Exon1-*Cyp4v3*^−/−^ mouse [5–7]. It analyzes various forms of nontransparent science in published papers on these *Cyp4v3*^−/−^ mouse models. Furthermore, this section discusses flaws of these *Cyp4v3*^−/−^ mice in modeling BCD.

Nontransparent science issues in these *Cyp4v3*^−/−^ mouse papers are discussed by analyzing critical data and information missing from the papers, self-conflicting data or data conflicting with 3rd party results, data presentation and quality issues, authors contributions, and/or editorial history of these papers, with references to journal policies and guidelines of relevant self-regulatory bodies. Data presented by the authors were taken at face value when analyzing animal model flaws.

### The Cyp4v3^-/-^ mouse

#### Nontransparent science

*Premature publication of an animal model without clinically significant data.* BCD is a blinding disease caused by severe retinal degeneration (Fig. 1). However, the *Cyp4v3*^−/−^ Mouse Paper did not include any clinically significant data demonstrating that the *Cyp4v3*^−/−^ mouse has retinal degeneration, abnormal retinal function or severe vision loss as seen in BCD [5]. Without such data, calling the *Cyp4v3*^−/−^ mouse a model of BCD in the paper title appeared to be premature. While the authors did state at the end of the paper that further detailed characterization of this model would be required to validate the *Cyp4v3*^−/−^ mouse as a BCD model, including electrophysiological changes via electroretinograms (ERG), spectral domain optical coherence tomography (SD-OCT), and optokinetic (OKN) measurements of visual acuity, for better transparency, this critical study limitation and the preliminary nature of the report should be highlight upfront in the paper in the abstract, or clarify in the title by adding phrases such as a preliminary or interim report instead of at the end of the paper.

The paper included data showing the *Cyp4v3* gene was knocked out in *Cyp4v3*^−/−^ mice, and reported crystal deposition in the eye and serum fatty acid abnormalities, with the latter also reported in BCD patients [34]. However, there were already signs that the *Cyp4v3*^−/−^ mouse do not phenocopy BCD in humans. For example, as reported in the paper, histologic sections of murine retinas showed that tissue structures appear normal in both *Cyp4v3*^−/−^ mice and WT mice. Further, the authors noted that C18:0 fatty acid abnormality in *Cyp4v3*^−/−^ mice was in the opposite direction to that reported in BCD patients. In light of this, it would be prudent for the authors to complete the study with the ERG, OCT and OKN results and then publish the paper. *Question*: Since the authors acknowledged that it is necessary to have ERG, OCT and OKN data to validate the *Cyp4v3*^−/−^ mouse as a BCD model, why didn’t the author wait till they have such data and then publish the paper instead of publishing a preliminary report?

*Not providing corrections when flaws to the model are discovered.* Whne critical data is missing from a preliminary research report, the authors have an obligation to update or correct the original publication with the missing data once it becomes available. In searching for visual function and retinal structure data after the *Cyp4v3*^−/−^ Mouse Paper was published, the firsth author later reported in her Ph.D. dissertation (with the correspondence author being her academic advisor and Chair of the Reading Committee of her dissertation) ERG, OCT, and OKN assessments conducted on this mouse. Based on ERG, OCT and OKN test results from 12 (middle age) and 18-month-old (old age) mice, the first author concluded on page 66 of her dissertation that *Cyp4v3*^−/−^ mice had similar visual function, retinal structure, and visual acuity compared to wild-type mice [35]. Because the authors stated in the *Cyp4v3*^−/−^ Mouse Paper that these tests (ERG, OCT and OKN) were necessary to validate the *mouse model*, the test results and conclusions actually invalidated the *Cyp4v3*^−/−^ mouse as a BCD model by the authors’ own standards. The journal which published the *Cyp4v3*^−/−^ Mouse Paper is in the field of ophthalmology and visual science. It requires authors to correct the science in a paper, and when the validity of the paper is sufficiently compromised, to retract the paper [36]. Since the validity of the *Cyp4v3*^−/−^ mouse model was not only compromised, but completely destructed even by the authors’ own standards, the authors should have informed the journal about the invalidating results and to retract the paper immediately. Moreover, the flaws of the *Cyp4v3*^−/−^ mouse model have been pointed out by various BCD research groups [6, 7, 37, 38]. However, the journal web page of the *Cyp4v3*^−/−^ Mouse Paper showed that no correction has been made to the paper, let alone retraction [39]. As a result, years after the invalidating data became known to the authors, the *Cyp4v3*^−/−^ Mouse Paper continues to mislead the public about the *Cyp4v3*^−/−^ mouse model. *Question*: Have the authors informed the journal about the ERG, OCT and OKN results of the *Cyp4v3*^−/−^ mouse later discovered by the authors and requested for a correction or retraction of the *Cyp4v3*^−/−^ Mouse Paper that was published earlier? If no, why?

#### Animal model flaws

*No vision loss or retinal degeneration.* As discussed above, at middle age (12-month) and even old age (18-month), retinal function (via ERG), visual acuity (via OKN) and retinal structure (via OCT) test results of the *Cyp4v3*^−/−^ mice were similar to those of WT mice [35]. This invalidated the *Cyp4v3*^−/−^ mouse as a model of BCD, a blinding disease caused by severe retinal degeneration in humans. In contrast, BCD patients reach legal blindness by middle age and ERG extinction occurs long before legal blindness [9, 15]. Severe retinal degeneration (including RPE atrophy) is also evident at middle age for BCD patients, e.g., see Fig. 1 A for OCT image of the author taken at the age of 43. Clearly, the *Cyp4v3*^−/−^ mice do not recapitulate the natural course of BCD either.

Moreover, minor phenotype seen in *Cyp4v3*^−/−^ mouse model mainly exhibits impairment of photoreceptors but not of RPE, whereas clinical findings in patients show that BCD affects the RPE first before the photoreceptors [5, 40]. This difference further demonstrates that *Cyp4v3*^−/−^ mouse is not an appropriate BCD model.

*Retinal crystals and serum fatty acid abnormalities*. The *Cyp4v3*^−/−^ Mouse Paper reported that the *Cyp4v3*^−*/*−^ mouse had retinal crystals and serum fatty acid abnormalities, with the latter also reported in BCD patients [5, 34]], Based on these findings, the authors described the *Cyp4v3*^−/−^ mouse as a model of BCD. However, crystal depositions have also been reported in lymphocytes and skin fibroblasts of BCD patients but do not cause any clinically significant abnormalities [41]. As a disease, clinically significant abnormalities of BCD remain only in the eye. The fact that retinal crystals were seen in *Cyp4v3*^−/−^ mice but these mice had no vision loss proved that retinal crystals in the mouse model cannot be used as a proxy or a surrogate biomarker for BCD. Further, retinal crystals are not unique to BCD and a variety of diseases or factors can result in retinal crystals, such as different types of crystalline retinopathy, chronic retinal detachment or drug use [18, 19]. A paper reported that retinal crystals can even be caused by high dose of lutein in healthy individual and do not affect vision [20]. Moreover, retinal crystals seen in *Cyp4v3*^*−/−*^ mice are different from those in BCD in various ways: (i) they do not appear in all *Cyp4v3*^−/−^ mice [6], (ii) the number of retinal crystals in *Cyp4v3*^−/−^ mice are less numerous as seen in BCD [5], and (iii) retinal crystals became more obvious in *Cyp4v3*^*−/−*^ mice over time [5], whereas retinal crystals start disappearing as BCD advances in human [11, 13, 16, 17].

Indeed, the fact that the *Cyp4v3*^−/−^ mouse copied the look of BCD (retinal crystals) but failed to show function loss (vision loss) reminded me of the infamous animal models of AD which also copied the hallmark characteristics of AD (amyloid plaques in the brain) but failed to show function loss (memory loss) of AD [42, 43]. The most commonly used AD animal models are transgenic mice that overexpress human genes associated with familial AD that result in the formation of amyloid plaques [43]. The high failure rate of AD clinical trials and waste of billions of dollars has been related to the premature translation of highly successful results in animal models that mirror only limited aspects of AD pathology to humans [26, 42, 43]. The overwhelming false positive rate and very poor clinical translatability of AD animal models shows the danger of using the *Cyp4v3*^−/−^ mouse as a BCD model and for screening BCD drug candidates in preclinical proof-of-concept (POC) studies.

Further, the serum fatty acid abnormalities reported in the *Cyp4v3*^−/−^ mice is not a clinically relevant phenotype of BCD either. Although serum fatty acid abnormalities have been reported in BCD patients, such abnormalities do not manifest as any systemic disease. As a disease, BCD only affects the eye. In fact, even the *Cyp4v3*^−/−^ Mouse Paper acknowledged that one of its study limitations was that although there is documented dyslipidemia in BCD patients, the clinical relevance has not yet been determined [5].

Finally, the *Cyp4v3*^−/−^ Mouse Paper noted that serum free fatty acid (FFA) abnormalities found in *Cyp4v3*^−/−^ mice are opposite in direction to those reported in BCD patients [5]. For example C18:0 in *Cyp4v3*^−/−^ mice was shown to be lower than controls, whereas the levels in BCD patients are higher than in healthy individuals. Similarly, C18.1, C18.2, and C18.3 also showed differences in *Cyp4v3*^−/−^ mice compared to BCD patients [5, 34], suggesting Cyp4v3 may play a different role in mice from that of CYP4V2 in humans.

*No proof of Cyp4v3 protein expression in WT mouse retina.* Two years before publishing the *Cyp4v3*^−/−^ Mouse Paper, the same research group published a paper showing that the CYP4V2 protein is expressed in human retina, which is the tissue affected in BCD [44]. The paper also discussed the importance of CYP4V2 in the retina and its enzymatic activities. In contrast, the *Cyp4v3*^−/−^ Mouse Paper did not include any data proving that like CYP4V2 protein expression in human retina, the Cyp4v3 protein is also expressed in WT mouse retina. Interestingly, knowing the critical role of the CYP4V2 protein in human retina, the authors only provided data showed that the cyp4v3 protein is expressed in WT mouse liver [5].

### The high fat diet (HFD)-Cyp4v3^-/-^ mouse

Independent of the study reported in the *Cyp4v3*^−/−^ Mouse Paper, the authors of the HFD-*Cyp4v3*^−/−^ Mouse Paper also developed the *Cyp4v3*^−/−^ mice [6]. Like what the authors of the *Cyp4v3*^−/−^ Mouse Paper found, the authors of the HFD-*Cyp4v3*^−/−^ Mouse Paper also found that that *Cyp4v3*^−/−^ mice have normal ERGs [6]. Further, the HFD-*Cyp4v3*^−/−^ Mouse Paper noted that the lack of retinal dysfunction (ERG phenotype) makes the *Cyp4v3*^−/−^ mouse an inappropriate BCD model for assessing gene therapy efficacy in preclinical studies. The HFD-*Cyp4v3*^−/−^ Mouse Paper reported that high-fat diet (HFD) induced retinal phenotypes in the *Cyp4v3*^−/−^ mouse, particularly reduced ERGs. Based on these HFD-induced phenotypes, the authors claimed the HFD-*Cyp4v3*^−/−^ mouse is an animal model for BCD.

#### Nontransparent science

*Lack of warning of critical study limitations.* HFD is known for causing various disorders in men and mice, with many reports of HFD inducing a wide range of retinal dysfunction and systemic abnormalities in WT mice, including ERG, retinal gene expression, and biochemical abnormalities, including alterations in serum fatty acid concentration [45–47]. In fact, HFD-WT mice have been used as an animal model for diabetic retinopathy (DR), a common retinal disease [45–47]. However, the HFD-*Cyp4v3*^−/−^ Mouse Paper did not state this critical study limitation. *Question*: Did the authors know that HFD can induce retinal dysfunctions including ERG and and other abnormalities in WT mice? If no, why did the paper omit large amount of data of the HFD-WT mouse control group (see discussion below)? If yes, why didn’t the author state this critical study limitation in the paper?

*Missing critical control data.* In addition, compared to the other mouse groups, a large amount of data related to HFD impact on WT mice was missing from the paper. For example, comparisons of all four mouse groups in the study were not presented at all tested timepoints (WT mice fed a normal diet (ND) [ND-WT] or HFD [HFD-WT], and *Cyp4v3*^−/−^ mice fed with ND [ND-*Cyp4v3*^−/−^] or HFD [HFD-*Cyp4v3*^−/−^]). Specifically, ERG data from HFD-WT mice, a critical control group for HFD-*Cyp4v3*^−/−^ mice, were only included at the 12-week timepoint, whereas data for all other groups were largely reported at various timepoints up to 20 to 24 weeks of age. Furthermore, an ERG time dependence profile was shown for all mouse groups except for the HFD-WT mouse group (Fig. 1g & 3b), preventing the comparison of HFD-*Cyp4v3*^*−/−*^ mouse group to HFD-WT mouse group beyond 12-weeks of age [6]. In addition to the missing ERG data, the RPE FFFA data of the HFD-WT mouse control group was also missing (Fig. 6 of the HFD-*Cyp4v3*^−/−^ Mouse Paper). *Questions*: When comparing the HFD-*Cyp4v3*^−/−^ mice to its control groups, why ERG data of the HFD-WT control group was missing in Fig. 3b and and RPE FFA data of the HFD-WT control group was missing from Fig. 6 of the HFD-*Cyp4v3*^−/−^ Mouse Paper? Can the authors provide these missing data so to give the readers a full picture of the relevant results?

Significantly, reduced ERGs in HFD-WT mice of the same strain (C57BL/6J) have been reported by other researchers during study periods overlapping with those in the HFD-*Cyp4v3*^−/−^ Mouse Paper [45, 46]^1^^,^^2^. One report has shown significantly reduced ERG in C57BL/6J WT mice fed with a HFD nearly identical to that used by the authors for two months, which are comparable to 12-week-old time point evaluated by the HFD-*Cyp4v3*^−/−^ Mouse Paper [46]. Reduced ERG have also been reported in C57BL/6J WT mice after 12 weeks and five months of HFD feeding, respectively, which are comparable to 16-week-old and 24-week-old time points evaluated by the HFD-*Cyp4v3*^−/−^ Mouse Paper [45, 46] (ERG data for 16-week, 20-week and 24-week-old HFD-WT mice were not shown in the HFD-*Cyp4v3*^−/−^ Mouse Paper) [6].

Collectively, the fact that ND-*Cyp4v3*^−/−^ mice have normal ERG and HFD-induced ERG abnormalities are seen in both WT mice and *Cyp4v3*^−/−^ mice suggests that the reduced ERGs seen in HFD-*Cyp4v3*^−/−^ mice are attributable to HFD and not to *Cyp4v3* knockout, thereby indicating that the HFD-*Cyp4v3*^−/−^ mouse is not an appropriate model for studying BCD.

*Selective disclosure.* The HFD-*Cyp4v3*^−/−^ Mouse Paper stated that to verify the fidelity of ERG in the HFD-*Cyp4v3*^−/−^ mice, the authors systematically analyzed and compared the results of four groups of mice, ND)-WT, ND-*Cyp4v3*^−/−^, HFD-WT, and HFD- *Cyp4v3*^−/−^ mice. The Materials and Methods section reveals that ERG tests were performed in mice up to 24-week-old [6]. However, the ERG results and comparison among the four mouse groups presented in the paper appears to be selective rather than systematic, which could affect validity of the HFD-*Cyp4v3*^−/−^ mouse as a BCD model. For example, besides missing HFD-WT mouse ERG data after 12-week-old, the paper showed HFD-*Cyp4v3*^−/−^ mouse ERG data only up to 20-week-old, as compared to up to 24-week-old ERG data shown for the other two mouse groups, the ND)-WT mice and the ND-*Cyp4v3*^−/−^ mice. Since ERG of the HFD-*Cyp4v3*^−/−^ mouse group already stabilized and even showed sign of improvement at 20-week-old when compared to the control groups, it would be interesting and important to see if ERG of the HFD-*Cyp4v3*^−/−^ mice continues to improve at 24-week-old, which would invalidate the HFD-*Cyp4v3*^−/−^ mouse as a BCD model because as a blinding disease like BCD patients’ ERGs decline over time till extinction rather than stabilize and improve over time. *Questions: For* a systematic comparison, can the authors show up to 24-week-old ERG data of all four mouse groups? Can the authors show proof of ERG extinction in the HFD-*Cyp4v3*^−/−^ mice?

*Questionable data quality – inconsistent animal numbers.* Besides showing ERG data of different mouse groups up to different timepoints, another strange thing about the ERG data presented in the HFD-*Cyp4v3*^−/−^ Mouse Paper is that the number of mice in each mouse group is inconsistent when the ERG data was compared to different mouse groups. For example, for the ND-*Cyp4v3*^−/−^ mouse control group, the number of mice were 28 when ERGs of this group was compared to those of the ND-WT mouse group up to 24-week-old. However, the number of the ND-*Cyp4v3*^−/−^ mice reduced to 20 when their ERGs were compared to ERGs of all other mouse groups, including the HFD-*Cyp4v3*^−/−^ mouse group up to 20-week-old. Similarly, the number of mice in the ND-WT mouse control group was 31 when their ERGs were compared to those of ND-*Cyp4v3*^−/−^ mouse group up to 24-week-old. However, the mouse number reduced to 27 when the ERGs of the ND-WT group were compared to all other mouse groups, including the HFD-*Cyp4v3*^−/−^ mouse group up to 20-week-old. (Figure 1f, 1g, 3a &3b of the HFD-*Cyp4v3*^−/−^ Mouse Paper). *Questions*: Can the authors explain why the number of mice in these two control groups (ND-WT and ND-*Cyp4v3*^−/−^) decreased when their ERGs were compared to those of the HFD-*Cyp4v3*^−/−^ mouse group? Can the authors show ERG data from all mice in these two control groups when comparing them to the HFD-*Cyp4v3*^−/−^ mouse group so that the readers can look at full results?

Further, among all mouse groups, the HFD-WT control group has the lowest number in ERG data shown (18 mice compared to 31, 28 and 26 mice for other groups). The paper did not state how many mice were enrolled in each group at the onset of the study. Thus, it is not clear if the ERG data presented in the paper was generated from all mice enrolled in each of the four mouse groups. *Questions*: Can the authors state clearly how many mice were enrolled in each of the four groups at the onset of the study? Was 18 the full number for the HFD-WT mouse group? If so, was it by chance that this group of which large amount of data was missing also have the lowest animal number among all groups?

**Fig. 2:**
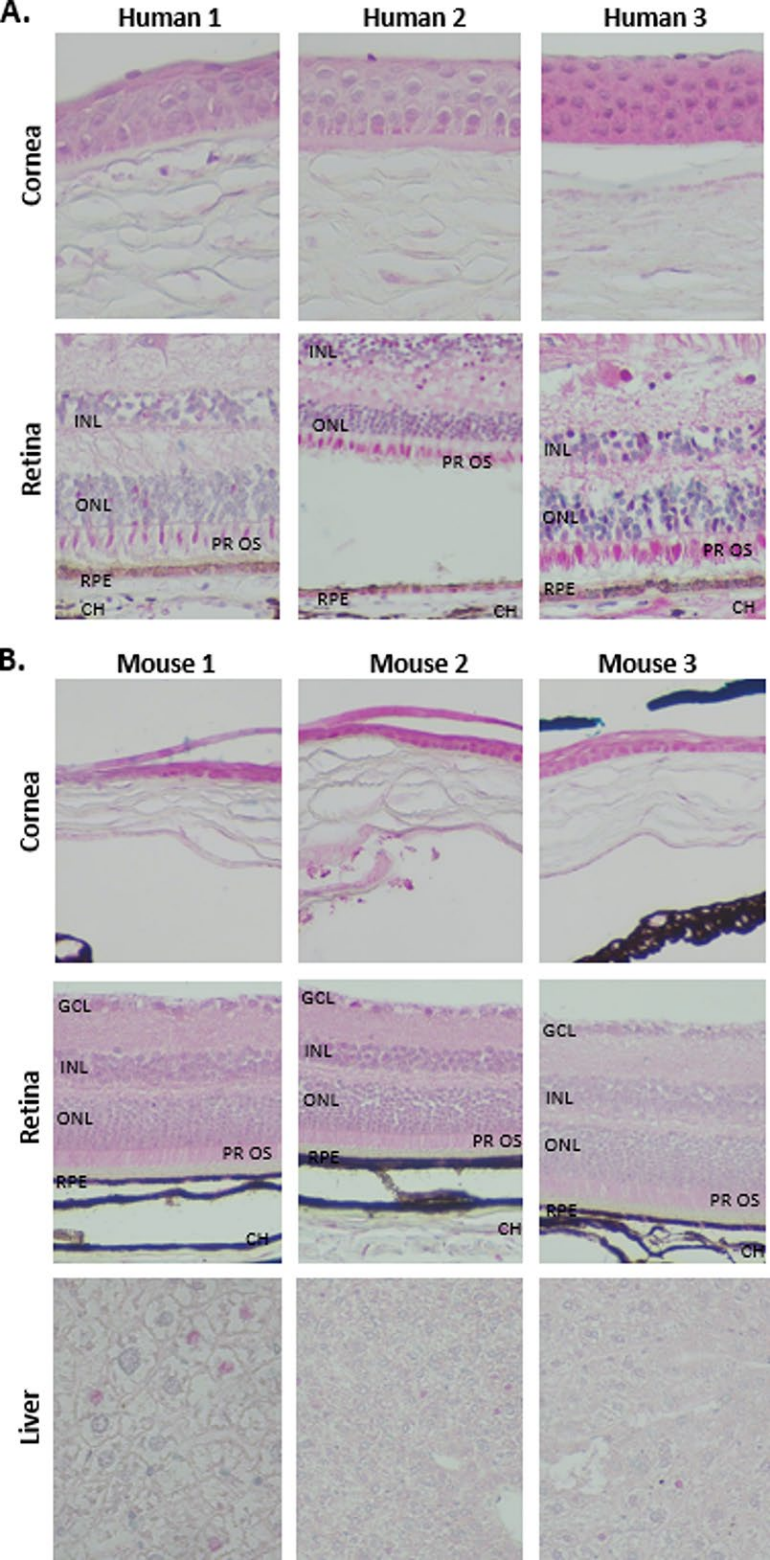
Marked difference between retinal expression of CYP4V2 protein in humans and Cyp4v3 protein in mice. (A) CYP4V2 immunohistochemical (IHC) staining in human retina and cornea, and (B) Cyp4v3 IHC staining in wild type (WT) C57BL/6J mouse retina, cornea and liver. Significantly, IHC staining reveals that unlike CYP4V2 protein expression in normal human retina, Cyp4v3 protein-specific positive staining is only observed in wild type mouse corneal epithelium, and is not expressed in any part of the mouse retina. In contrast, human eye showed CYP4V2 protein-specific positive staining in the RPE; cone outer segments; occasional nuclei in the inner and outer nuclear layers, and the corneal epithelium. The markedly different retinal expression profile between mouse and human retina suggests that unlike CYP4V2 protein in human retina, the murine ortholog Cyp4v3 protein does not play a role in the retina of wild type mice. This indicates that the *Cyp4v3* knockout mouse (whether fed with normal diet (ND) or high-fat diet (HFD), and regardless of the knockout strategy) is not an appropriate model for BCD, a human retinal disease resulting from a lack of normal retinal CYP4V2 protein expression. GCL = ganglion cell layer; IPL = inner plexiform layer; INL = inner nuclear layer; ONL = outer nuclear layer; PR OS = photoreceptor outer segment; RPE = retinal pigment epithelium; CH = choroid. Brown/black staining is natural melanin, blue is hematoxylin counterstain, and bright pink/red is antibody positivity (CYP4V2 for human, Cyp4v3 for mouse). Magnification of all images at 20X. Cross reactivity of the anti-human CYP4V2 antibody to the mouse ortholog Cyp4v3 is confirmed by positive staining in the livers and corneal epithelium of WT mice

The inconsistent animal number issue also appears in other dataset. For example, Fig. 2d of the HFD-*Cyp4v3*^−/−^ Mouse Paper included data from 28 ND-*Cyp4v3*^−/−^ mice which was the maximum number for this group as shown in ERG dataset. In contrast, only 11 out of 26 mice in the HFD-*Cyp4v3*^−/−^ mouse group was used for comparison. *Questions*: Can the authors please show full results of all 26 mice of the HFD-*Cyp4v3*^−/−^ mouse group in Fig. 2d? how can results from 11 out of 26 mice represent the whole group?

*Questionable data quality - nonsense error bars.* Moreover, the ERG data in the HFD-*Cyp4v3*^−/−^ Mouse Paper have very large error bars for all mouse groups, even for the WT mouse group. The authors did not explain why even the ND-WT mice in the study have shown such high variability of ERG. According to the legend of various ERG figures throughout the HFD-*Cyp4v3*^−/−^ Mouse Paper, e.g., Fig. 1f, 1g, 3a & 3b, the error bars indicate the standard error of the mean (s.e.m.). Given the sample size (n) of the four mouse groups ranges from 18 to 31, the ERG error bars of all mouse groups (including the WT mouse group), if shown in standard deviation (SD) would be about 4–5 times in length of the error bars presented in the paper (indicating s.e.m) and run off chart. This does not seem to make sense. *Questions*: can the authors explain why their mice have such high variability in ERG data, even including the WT mice? Are results wwith such high variability still reliable?

*Questionable representative image – self-conflicting data.* Representative fundus images of a HFD-*Cyp4v3*^−/−^ mouse (Fig. 4d of the HFD-*Cyp4v3*^−/−^ Mouse Paper) before gene therapy injection (self-complementary adeno-associated virus 8 expressing human *CYP4V2* [AAV8-hCYP4V2]) showed retinal lesion. Since the gene therapy injections were done at 4-weeks of age (Fig. 4a), fundus images of HFD-*Cyp4v3*^−/−^ before injection must have been taken when the mice were 4 weeks old or younger. However, according to Fig. 2d of the paper, retinal lesions in HFD-*Cyp4v3*^*−/−*^ mice do not appear until 5 weeks, with less than 40% developing retinal lesions even by 6 weeks of age. Thus, the retinal lesions shown in Fig. 4d before injection (4 week-old or younger) seem to conflict directly with results presented by the authors in Fig. 2d which indicates that 0% of the HFD-*Cyp4v3*^−/−^ mice had retinal lesion at 4-week-old or yonder. Further, I was told that the gene therapy treatment images before injection and 18 days after injection (bottom left and bottom right, respectively) in Fig. 4d of the HFD-*Cyp4v3*^−/−^ Mouse Paper do not appear to be images taken from the same eye. This indicates another flaw in the result shown in Fig. 4d. 18 days after injection at 4-week-old is equivalent of mice between 6 and 7 weeks old. According to Fig. 2d, about 50% of the HFD-*Cyp4v3*^−/−^ mice have not shown retinal lesion at this time point. Therefore, unless the gene therapy treatment images (both before and after injection) in Fig. 4d showed retinal lesion disappearing after gene therapy in the same eye, the authors cannot use the 18 days after injection image on the bottom right to show gene therapy efficacy because even without gene therapy treatment, about half of the HFD-*Cyp4v3*^−/−^ mice did not have retinal lesion at this age. How did the author know if the lack of retinal lesion was a result of gene therapy? Even if the bottom left and right images were taken from the same eye, as discussed above, the before injection image at the bottom left should have shown no retinal lesion. Therefore, Fig. 4d cannot be used to show gene therapy efficacy in the HFD-*Cyp4v3*^−/−^ mice. *Questions*: Can the authors explain why the representative image (before injection in Fig. 4d) showed retinal lesion but according to Fig. 2d, it should have 0% chance to show retinal lesion? Were the bottom left and bottom right images in Fig. 4d taken from the same eye of the same mouse?

As analyzed above, the HFD-*Cyp4v3*^−/−^ Mouse Paper has a series of nontransparent science issues. Just to name a few, it failed to disclose that HFD also causes retinal dysfunctions in WT mice, showed ERG data with nonsense error bars (Fig.  1f, 1g, 3a & 3b), showed partial results with omitted data points (e.g., ERG data from reduced number of animals for the ND-WT and ND-*Cyp4v3*^−/−^ control groups (Fig. 3a and b), missing ERG and RPE FFA data for the HFD-WT control group in Figs. 3b and 6)), and showed a representative image (Fig. 4d) which contradicts the authors’ own result (Fig. 2d), and showed big variability in the number of mice among the four mouse groups (ranging from 18 to 31) when presenting the ERG data without explaining why or stating what was the full size of each group. Further, the HFD-*Cyp4v3*^−/−^ mice do not go blind. Their ERGs do not decline continuously like in BCD. These issues significantly undermine the validity of the HFD-*Cyp4v3*^−/−^ mouse as a BCD model and efficacy of the gene therapy in such mouse model. Additionally, some of these data presentation patterns could cause concerns about data quality [48].

Some glaring issues such as missing critical control data, comparison of ERG data of different mouse groups up to different timepoints, inconsistent animal numbers, and shockingly large error bars (even for WT mice) in the HFD-*Cyp4v3*^−/−^ Mouse Paper can be spotted by anyone with basic training in natural science even without background in biomedical research or ophthalmology. In contrast, the HFD-*Cyp4v3*^−/−^ Mouse Paper was not only published, but also received speedy acceptance in the peer review process and was even recommended by the editor. The paper was published at an incredible speed of less than two months from manuscript submission (Received 6 April 2020; Accepted 19 May 2020; Published 1 June 2020). From submission to acceptance for publication, the paper only took 44 days, which is just a quarter of the average time of 172 days from submission to acceptance for the journal. The journal web page of the HFD-*Cyp4v3*^−/−^ Mouse Paper shows that the paper was even picked as the Editor’s Choice [49, 50]. *Questions*: During the manuscript review process, have the editor and reviewers expressed concerns about these nontransparent science issues in the HFD-*Cyp4v3*^−/−^ Mouse Paper? What criteria was used in picking it as the Editor’s Choice? Have the editor and reviewers for the HFD-*Cyp4v3*^−/−^ Mouse Paper been screened to avoid potential conflict of interest?

#### Animal model flaws

The HFD-*Cyp4v3*^−/−^ Mouse Paper stated that a knockout animal model needs to faithfully represent the human disease for use in the evaluation of potential therapy [6]. However, the HFD-*Cyp4v3*^−/−^ mouse failed to faithfully mimic BCD in human patients.

*Retinal phenotypes were attributable to HFD but not Cyp4v3*^*−/−*^. As analyzed above, ERG phenotype seen in HFD-*Cyp4v3*^−/−^ mice was likely caused by HFD which also causes reduced ERG in WT mice, but not due to *Cyp4v3* knockout. This indicates that the HFD-*Cyp4v3*^−/−^ mouse is not an appropriate model for studying BCD.

*No ERG extinction - ERG stabilized and improved over time.* Importantly, even after HFD stress, the HFD-*Cyp4v3*^−/−^ mice still did not go blind. Figure 3b of the HFD-*Cyp4v3*^−/−^ Mouse Paper shows that ERGs of HFD-*Cyp4v3*^−/−^ mice did not continue to decline but stabilized and even showed sign of improvement over time after an initial decline [6]. The differences to the control groups narrowed at 20-week-old time point. This is dramatically different to the continuous decline of ERG, till extinction, and decrease in visual acuity observed in BCD [9, 12, 15, 16]. Interestingly, the paper stop showing ERG data of the HFD-*Cyp4v3*^−/−^ mice at the 24-week-old time point despite ERGs were conducted up to 24-week-old and ERGs of two other mouse groups (ND-WT and ND-*Cyp4v3*^−/−^) were shown and compared up to 24-week-old time point (Fig. 1 of the HFD-*Cyp4v3*^−/−^ Mouse Paper).

*No obvious retinal degeneration*. Significantly, the OCT and histology hematoxylin and eosin staining (H&E staining) images of the HFD-*Cyp4v3*^−/−^ mouse retinas did not show severe retinal degeneration as seen in BCD. BCD patients have severe RPE atrophy with loss of the outer retina (Fig. 1), which is the clinical cause of blindness in these patients [10–13, 40]. In contrast, the HFD-*Cyp4v3*^−/−^ Mouse Paper only showed retinal lesions in HFD-*Cyp4v3*^−/−^ mice, not the typical severe RPE atrophy and retinal degeneration associated with BCD. Retinal lesions are not a characteristic of BCD and are not unique to HFD-*Cyp4v3*^−/−^ mice, as HFD also causes retinal lesions in WT mice [51].

Blindness and retinal degeneration are the two most important clinical phenotypes of BCD. They are what we, the patients, care most about. Like the *Cyp4v3*^−/−^ mouse, the HFD-*Cyp4v3*^−/−^ mouse failed to mimic either of them as seen in BCD patients. This gives rise to issue of pharmacological relevance and clinical translatability using the HFD-*Cyp4v3*^−/−^ mouse in preclinical POC studies to test potential drug candidates for treating BCD.

Several other endpoints have been described by the HFD-*Cyp4v3*^−/−^ Mouse Paper as demonstrating either consequence of HFD on BCD phenotype or efficacy of a gene therapy in the *Cyp4v3*^*−/−*^*-*HFD mouse model.

*Retinal thinning pattern different from BCD.* The paper reported that HFD-*Cyp4v3*^−/−^ mice had increased retinal thickness following treatment with adeno-associated virus (AAV)-8-hCYP4V2 compared to a control vector expressing green fluorescent protein (eGFP) (AAV8-eGFP). However, retinal thinning as a phenotype was not established in HFD-*Cyp4v3*^−/−^ mice because no data was provided in comparison to control groups. As shown in H&E staining image, changes in retinal thickness in AAV8-eGFP group compared to AAV8-CYP4V2 treatment group did not show retinal degeneration. The representative H&E staining image of the HFD-*Cyp4v3*^−/−^ mouse retinae (Fig. 5g of the HFD-*Cyp4v3*^−/−^Mouse Paper) showed that the thickening of the retina following AAV8-hCYP4V2 vector treatment is mainly attributed to excessive thickening of the inner plexiform layer (IPL) when compared to the retina treated by the control vector. IPL thinning in the HFD-*Cyp4v3*^−/−^ mouse is a different phenotype than the loss of the outer retina and severe RPE atrophy that occurs with BCD patients (Fig. 1).

*No retinal crystals.* The HFD-*Cyp4v3*^−/−^ mouse did not display retinal crystals, which is a non-pathological phenotype of BCD. Contrary to the fine, shiny crystals seen in BCD patients, large, patchy retinal lesions are seen in HFD-*Cyp4v3*^−/−^ mice. These lesions are similar to those seen in HFD-WT mice, but are not representative of the type of crystals seen in BCD (Fig. 1) [51].

*RPE FFA profile.* The RPE FFA profile for ND-WT, ND-*Cyp4v3*^*−/−*^, HFD-*Cyp4v3*^*−/−*^ mice, and HFD-*Cyp4v3*^−/−^ mice treated with an experimental gene therapy was shown in Fig. 6 of the HFD-*Cyp4v3*^*−/−*^ Mouse Paper. Notably, the paper did not provide the FFA profile for the HFD-WT mouse control group. Importantly, although a significant increase in one of the examined FFA was demonstrated following gene therapy (FFA18.3), this FFA was not decreased in ND-*Cyp4v3*^*−/−*^ mice nor was it shown that this FFA was significantly decreased in HFD-*Cyp4v3*^*−/−*^ mice compared to ND-WT mice (HFD-WT data not shown). There were no changes in any other examined FFA except that (i) the gene therapy decreased the normal level of FFA18:1 in HFD-*Cyp4v3*^*−/−*^ mice and (ii) HFD feeding reduced FFA20:4 level in *Cyp4v3*^*−/−*^ mice to the normal level seen in ND-WT mice but gene therapy increased it.

*No proof of Cyp4v3 protein expression in WT mouse retina.* Like the *Cyp4v3*^−/−^ Mouse Paper, the HFD-*Cyp4v3*^−/−^ Mouse Paper also failed to show that the Cyp4v3 protein is expressed in WT mouse retina. Only Southern blot result was shown for WT mouse and the sample used for genotyping was tail tip [6].

A comparison of the retinal phenotypes of the *Cyp4v3*^−*/*−^ mouse and the HFD-*Cyp4v3*^−/−^ mouse models to human BCD is summarized in Table 1.

**Table 1:** Comparison of *Cyp4v3*^−/*−*^ and HFD-*Cyp4v3*^−/−^ mouse models to BCD in humans

Model Phenotypes	BCD in humans	*Cyp4v3*^−/−^ mouse^5^	HFD-*Cyp4v3*^−/−^ mouse^6^
**Blindness**	**Yes**	**No**	**No**
	Visual function (ERG pathologic findings):^15^ - Early stage (preceding loss of central vision): diminished ERG. - Intermediate stage (long before legal blindness occurrence): Extinction of ERG occurs already. Visual acuity^9,12,16^ - Continuous decline till legal blindness.	Visual function (ERG): normal - 24-week-old^6^ - 12- and 18-month-old^35^ Visual acuity (OKN): normal - 12- and 18-month-old: normal^35^	Visual function (ERG): unlike in BCD. - No ERG extinction - ERG stabilized after initial decline and improved over time, - Reduced ERGs were also reported in WT-HFD mice^45,46^.
**Severe retinal degeneration**	**Yes**	**No**	**No**
	Retinal structure (OCT, FAF and other imaging modalities^10–14^, *see also* Fig. 1): - Extensive RPE atrophy - Loss of the outer retina. - Pigment clumps - Near total degeneration of all functional elements of the retina leaving only structural astrocytes.	Retinal structure: normal - OCT: 12- and 18-month-old^35^ - Histology: retinal tissue structures^5^.	Retinal structure (OCT and histology): - No evidence of extensive RPE atrophy or severe retinal degeneration as seen in BCD. - Retinal lesions are not unique to HFD-*Cyp4v3*^−/−^ mouse. HFD also causes retinal lesions in WT mice^51^.
**Retinal crystals**	**Yes**	**Yes but unlike in BCD**	**No**
	Retinal crystals^11,13,16,17^ - Numerous. - Disappearing in advanced stage. - Are not unique to BCD. A variety of diseases, such as other types of crystalline retinopathy, chronic retinal detachment, or even high dose of tamoxifen or lutein can cause retinal crystals^18–20^	Retinal crystals - Non-pathological: do not affect vision. - Are not unique to BCD^18–20^. - Not in all mice^6^ - Less numerous than in BCD. - Do not disappear in advanced stage.	Retinal crystals - No retinal crystals. - only large, patchy lesions were observed, similar to the lesions seen in HFD-WT mice^51^

Table legends: *Cyp4v3*^−/−^ mouse: *Cyp4v3* knockout mouse. ERG: electroretinograms. FAF: Fundus autofluorescence. HFD: high-fat diet. OCT: Optical coherence tomography. OKN: Optokinetic response. RPE: retinal pigment epithelium. WT: wild-type. Superscripted numbers indicate the corresponding reference listed in References

### The Exon1-Cyp4v3^-/-^ mouse

The Exon1-*Cyp4v3*^−/−^ Mouse Paper reported a novel mouse model of BCD generated by knocking out exon 1 of *Cyp4v3* in mice [7]. The Exon1-*Cyp4v3*^−/−^ Mouse Paper pointed out some defects of the *Cyp4v3*^−/−^ mouse and the HFD-*Cyp4v3*^−/−^ mouse models, e.g., that retinal degeneration and changes in ERG had no significant differences in the natural course of *Cyp4v3*^*−/−*^ mice and for the HFD-*Cyp4v3*^*−/−*^ mouse model, mice must be administered a high-fat diet. The paper reported that the Exon1-*Cyp4v3*^*−/−*^ mice showed various phenotypes of BCD, including reduced ERG and retinal degeneration. The authors of the Exon1-*Cyp4v3*^−/−^ Mouse Paper attributed the stronger phenotypes seen in Exon1-*Cyp4v3*^−/−^ mouse but not seen in the *Cyp4v3*^−/−^ mice developed in previous studies by other researchers to different gene knockout strategies. Specifically, the paper stated that both *Cyp4v3*^*−/−*^ mouse models in the *Cyp4v3*^*−/−*^ Mouse Paper and the HFD-*Cyp4v3*^*−/−*^ Mouse Paper deleted the entire coding region (26 kb and 28 kb, respectively) of the *Cyp4v3* gene, whereas the Exon1-*Cyp4v3*^*−/−*^ mouse was developed by using CRISPR/Cas9 to knock out only the entire exon 1 of *Cyp4v3*. However, the stronger phenotypes of the Exon1-*Cyp4v3*^*−/−*^ mouse over those of *Cyp4v3*^*−/−*^ mice are surprising and somewhat mysterious. Many questions are yet to be answered before the results can be relied upon.

#### Nontransparent science

*Surprising results over prior studies without a sound explanation.* Despite different knockout strategies, the Exon1-*Cyp4v3*^−/−^ mouse was expected to have the same protein consequence as the *Cyp4v3*^−/−^ mice generated by two different research groups in prior studies reported in the *Cyp4v3*^−/−^ Mouse Paper and the HFD-*Cyp4v3*^−/−^ Mouse Paper [5, 6]. In fact, the authors of the Exon1-*Cyp4v3*^*−/−*^ Mouse Paper acknowledged that (i) all these three knockout mice of *Cyp4v3* were generated from the same mouse strain (C57BL/6J), (ii) the Cyp4v3 protein is very simple and has no other isoforms, and (iii) their exon 1 knockout results in a failure in translation initiation of the entire Cyp4v3 protein [7]. In other words, whether knocking out only exon 1 or the entire *Cyp4v3*, both the Exon1-*Cyp4v3*^*−/−*^ mouse and the *Cyp4v3*^*−/−*^ mouse share the same protein consequence, that is, failure to express the entire Cyp4v3 protein. Hence, the Exon1-*Cyp4v3*^−/−^ mouse was expected to show no material retinal phenotype just like the *Cyp4v3*^−/−^ mouse. However, the authors of the Exon1-*Cyp4v3*^−/−^ Mouse Paper reported stronger phenotypes in the Exon1-*Cyp4v3*^−/−^ mouse than those of the *Cyp4v3*^−/−^ mice. The fact that *Cyp4v3*^−/−^ mouse had no material phenotypes was confirmed by two independent studies. Therefore, the stronger phenotypes reported in the Exon1-*Cyp4v3*^−/−^ mouse is rather surprising. However, the authors of the Exon1-*Cyp4v3*^*−/−*^ Mouse Paper failed to provide any scientific explanation, not even a hypothesis, as to why their Exon1-*Cyp4v3*^*−/−*^ mouse showed stronger phenotypes than the *Cyp4v3*^*−/−*^ mice previously developed by two different research groups and reported independently. This makes the Exon1-*Cyp4v3*^−/−^ Mouse Paper a mysterious report with surprising results.

The journal which published the Exon1-*Cyp4v3*^*−/−*^ Mouse Paper is specialized in disease models and mechanisms. As such, for transparent science, it is reasonable to expect the Exon1-*Cyp4v3*^*−/−*^ Mouse Paper to provide the mechanism to explain its surprising results over two previous studies. It is disappointing to see this mysterious report was published without a mechanism, an explanation or even a hypothesis to support its surprising results.

*Irrational decision.* Knowing that *Cyp4v3*^*−/−*^ mice developed in previous studies failed to mimic BCD, the authors of the Exon1-*Cyp4v3*^*−/−*^ Mouse Paper still chose a knockout strategy with the same protein consequence that was expected to repeat the same failure of previous *Cyp4v3*^*−/−*^ mouse models does not seem to make logical sense. Public funding for rare disease research is scarce. Why did the author choose to spend the precious funding on a strategy that was predicted to fail? Instead of knocking out exon 1, they could have knocked out a different exon which, unlike exon 1 knockout, would have a different protein consequence from the knockout of entire *Cyp4v3* strategy in previous studies. Such non-exon1 knockout strategy with a different protein consequence may have a better chance of generating phenotype results different from *Cyp4v3*^*−/−*^ mice. In addition, such strategies to knock out or create a mutation in non-exon1 locations could also better recapitulate the mutation region common in human patients, e.g., c.802-8_810del17insGC (exon 7), c.992 A > C (exon 8), and c.1091-2 A > G (intron 8) [52]. *Questions*: Can the authors explain why they bet their time and funding on a design that was predicted to generate the same protein consequence as prior *Cyp4v3*^−/−^ mouse models which failed?

*Failure to rule out non-Cyp4v3 factors which may contribute to surprising results.* In light of the surprising phenotypes seen in the Exon1- *Cyp4v3*^*−/−*^ mice but not seen in *Cyp4v3*^*−/−*^ mice developed by other researchers, the authors should have immediately thought of the possibilities of CRISPR/Cas9 off-target editing in other gene(s) and/or inherent or spontaneous mutations in the fertilized eggs of C57BL/6J mice used in their study, which could have contributed to the surprising phenotypes but not related to *Cyp4v3.* The authors should have conducted experiments and provided results in the paper to rule out these possibilities. However, the Exon1-*Cyp4v3*^*−/−*^ Mouse Paper did not even mention this obvious possibility, letting alone providing the relevant results. Hence, the paper failed to rule out the possibility that the stronger phenotypes seen in the Exon1- *Cyp4v3*^*−/−*^ mouse were associated with mutation(s) in gene(s) beyond *Cyp4v3* or were a result of interactions of such mutations(s) with *Cyp4v3*^*−/−*^ in mice. Consequently, the paper failed to establish that the phenotypes seen in the Exon1-*Cyp4v3*^*−/−*^ mice are exclusively attributable to *Cyp4v3*^*−/−*^.

The paper stated that the target ending with NGG near exon 1 of *Cyp4v3* was designed and cut under the action of CRISPR/Cas9, and the sequence of single guide (sg)RNA1 was 5′-CCGGCAGCGACTGGTCGCCACCT-3′, and the sequence of sgRNA2 was 5′-TCCGTCTACTACTCTAACTAAGG-3′. Because the CRISPR/Cas9 protospacer adjacent motif (PAM) NGG is missing from the sgRNA1 sequence. I assume the sgRNA1 sequence actually used by the authors was 5′-AGGTGGCGACCAGTCGCTGC CGG-3′ instead, which is the reverse complementary to the sgRNA1 sequence stated in the Exon1-*Cyp4v3*^*−/−*^ Mouse Paper. Interestingly, these two sgRNAs only have mediocre off-target scores (48 and 44, respectively) on Benchling, a popular tool for CRISPR guide RNA design. Both gRNAs scored below the off-target score threshold of 50 above which are considered as good guide [53]. This suggests that it is possible that these two sgRNAs may result in off-target editing in gene(s) besides *Cyp4v3* in mice. *Questions*: Have the authors thoroughly ruled out the possibilities of off-target editing in other gene(s)? Why didn’t the authors even mention this possibility in the paper? Do the authors have whole genome sequencing (WGS) comparison between the Exon1- *Cyp4v3*^−/−^ mouse and WT mice? Can the authors confirm what are the correct sequences of their gRNA1 and gRNA2?

Inherited or spontaneous mutations in other gene(s) could also contribute to the surprising phenotypes in the Exon1-*Cyp4v3*^*−/−*^ mice. For example, the *Crb1/Rd8* mutation is present in the C57BL/6N mouse substrain, which is used widely to produce knockout mice [54]. Presence of *rd8* can produce significant ocular disease phenotypes unrelated to the gene or genes of interest. It is suggested that researchers screen for *rd8* if their mouse lines were generated on the C57BL/6N background, bear resemblance to the *rd8* phenotype, or are of indeterminate origin [54]. Some vendors may sell the C57BL/6N strain (which have retinal phenotypes) even if the order is for the C57BL/6J strain because C57BL/6J strain is available only at certain vendors [55]. The possibility of retinal phenotype causing mutation(s) exist in WT mice is not remote. We learned this lesson in a hard way. A wrong WT mouse strain (C57BL/6N) was used by mistake in the ocular tolerability study of our BCD gene therapy product candidate even though the study protocol stated that C57BL/6J strain should be used. The 6N WT mice developed innate retinal phenotypes. Upon checking, these 6N mice had *Crb1* mutations which cause retinal abnormalities. Consequently, the ocular tolerability study had to be repeated using C57BL/6J WT mice ordered from the Jackson Laboratory to ensure they were of true 6J origin. Unlike the *Cyp4v3*^*−/−*^ Mouse Paper and the HFD- *Cyp4v3*^*−/−*^ Mouse Paper, the Exon1-*Cyp4v3*^*−/−*^ Mouse Paper did not disclose the source/vendor of the C57BL/6J mice or fertilized egg used in the study. Besides inherited mutations such as *rd8*, spontaneous mutations can also occur, including in the C57BL/6J mouse strain after certain generations of breeding [55]. An unintended mutation (*Crb1* or another retinal phenotype causing mutation), if exists in the Exon1-*Cyp4v3*^*−/−*^ mouse, can cause it to show retinal phenotypes that are not caused by *Cyp4v3* knockout and contribute to the surprising results. *Questions*: Can the authors disclose the source/vendor of the C57BL/6J control mice and fertilized egg used for CRISPR gene editing in the study? Can the authors confirm that the C57BL/6J mice used as control in the study and the fertilized eggs used for CRISPR/Cas9 gene editing were of the same parental line from the same vendor? Do the authors have sequencing results to confirm their Exon1- Cyp4v3^−/−^ mice do not have mutations in any other mouse gene that is known to cause retinal phenotypes?

*Lack of sufficient detail for others to replicate study and verify results.* The journal which published the Exon1-*Cyp4v3*^−/−^ Mouse Paper requires manuscripts to include in the materials and methods section sufficient detail to understand and replicate the experiments performed, as well as to provide names for all equipment and reagent suppliers [56]. However, besides the wrong sgRNA1 sequence and not disclosing the source/supplier of the C57BL/6J fertilized eggs, the paper also failed to identify the source/supplier of the sgRNAs and Cas9 mRNAs. In addition, the transfection protocol was described very briefly in a theoretical manner, simply stating that the sgRNA and Cas9 mRNAs were mixed by a certain concentration and proportion with a microinjection instrument. The paper did not state the concentration of the sgRNAs and Cas9 mRNA, the ratio between them or other experimental details [7]. In comparison, the HFD-*Cyp4v3*^−/−^ Mouse Paper provided a detailed description about the supplier and preparation of the sgRNA and Cas9 reagents, the microinjection protocol, as well as source/vendor of the C57BL/6J mice for generating the HFD-*Cyp4v3*^−/−^ mice [6]. Lack of sufficient details for others to replicate the study gives rise to concerns about results reproducibility. Taking together with the surprising results over prior studies without a sound explanation, this may affect credibility of the work. *Questions*: Can the authors fulfil the journal requirement and provide a detailed protocol for others to replicate the study and verify the results?

*Dubious authorship and study accountability.* The Author Contributions section of the Exon1-*Cyp4v3*^−/−^ Mouse Paper reveals that a co-correspondence author (who is also the last author hence presumably the study leader) was not involved in conceptualization, methodology, formal analysis, investigation, or writing the original draft of the paper [7]. Such senior and correspondence author was only involved in reviewing/editing of the manuscript. According to the International Committee of Medical Journal Editors (ICMJE) guideline referenced by the journal policies, an author must have made substantial contributions to the conception or design of the work; or the acquisition, analysis, or interpretation of data for the work in addition to drafting the work or revising it critically for important intellectual content [57, 58]. According to another guideline document referenced by the journal policies, acquisition of funding, the collection of data, or general supervision of the research group, by themselves, do not justify authorship. [57, 59]. Hence, pursuant to the ICMJE guideline, the last named author does not qualify as an author, let alone a correspondence author plus the last author which usually confers the role as the study leader or principal investigator. A guideline paper referenced by the journal policies stated that many people (both editors and investigators) feel that honesty in reporting science should extend to authorship. The rationale is that, if scientists are dishonest about their relationship to their work, this undermines confidence in the reporting of the work itself. [59]. *Question*: Given the study leader’s involvement in the rsearch was limited to reviewing/editing of the manuscript drafted by others, how can readers trust that the overall science and results of the paper? This is particularly troublesome in light of various forms of nontransparent science in the paper.

Each of the nontransparent science issues discussed above causes concerns about the results and/or conclusions of the paper. When all of them appear in one paper, it would be premature and imprudent to rely on its results before (i) authorship correction, (ii) rigorous verification of reproducibility after experimental details are provided, and (iii) performing tests (e.g., whole genome sequencing) to completely rule out potential non-*Cyp4v3* factors which might have contributed to the results (e.g., off-target editing and inherent or spontaneous mutations in the mouse strain).

#### Animal model flaws

As noted above, it would be premature to rely on the results of the Exon1-*Cyp4v3*^*−/−*^ Mouse Paper. For now, however, a preliminary review of its results already revealed the following animal model flaws.

*No ERG extinction.* The ERG changes in the Exon1-*Cyp4v3*^*−/−*^ mice are milder than in BCD patients. No ERG extinction was observed in Exon1-*Cyp4v3*^*−/−*^ mice, even at middle age (12-month). In contrast, BCD patients reach legal blindness by middle age and ERG extinction occurs long before legal blindness [9, 15].

*Different mRNA expression profile of CYP4V2 in human retina and Cyp4v3 in WT mouse retina.*Figure 1b of the Exon1-*Cyp4v3*^−/−^ Mouse Paper presented data on *Cyp4v3* mRNA expression in WT mouse retina. Because CYP4V2 functions as an enzyme [44], it is important to show Cyp4v3 protein expression rather than mRNA expression in WT mouse retina and compare to CYP4V2 protein expression profile in normal human eye. However, the paper did not provide evidence of Cyp4v3 protein expression in WT mouse retina. mRNA expression is not sufficient to predict protein expression levels [60].

In fact, even at the mRNA level, *Cyp4v3* expression in WT mice already showed dramatic difference from *CYP4V2* mRNA expression pattern in human retina. In human eyes, *CYP4V2* mRNA level in the retina (PLIER value of 93.78) is higher than that in the RPE/choroid (84.16) [61, 62]. In contrast, Fig. 1b of the Exon1-*Cyp4v3*^−/−^ Mouse Paper showed that Cyp4v3 mRNA level in WT mouse RPE/choroid complex is much higher (about 10 folds) than in WT mouse retina. This dramatic difference between human *CYP4V2* and mouse *Cyp4v3* mRNA expression pattern in the retina and RPE undermines the foundation for using mice to study BCD.

Further, the Exon1-*Cyp4v3*^−/−^ Mouse Paper only showed *Cyp4v3* mRNA expression level in WT mouse RPE/choroid complex, but not in RPE alone. *Cyp4v3* mRNA is expressed in mouse RPE/choroid complex does not necessarily mean it is expressed in mouse RPE. It is possible that the *Cyp4v3* mRNA detected in mouse RPE/choroid was largely contributed by mRNA expressed in the choroid but not from the RPE. Can the authors use assays such as RNAScope to pinpoint *Cyp4v3* mRNA expression localization in mouse choroid vs. RPE?

### Marked difference between retinal expression of CYP4V2 protein in humans and Cyp4v3 protein in WT mice

Clinically, outer retinal and RPE atrophy is a prominent feature of BCD and results in visual loss. Genetically, BCD is caused by *CYP4V2* mutations. The CYP4V2 mRNA and protein are expressed in normal human retina, including RPE cells [9, 44]. In fact, the same research group of the *Cyp4v3*^*−/−*^ Mouse Paper had previously presented data showing CYP4V2 protein expression in human retina in another paper [44]. Therefore, proof showing the Cyp4v3 protein is expressed in wild type (WT) mouse retina in the same pattern as the CYP4V2 protein is expressed in normal human retina is a pre-requisite for using *Cyp4v3*^*−/−*^ mice to study BCD. However, none of the *Cyp4v3*^−/−^ Mouse Paper, the HFD-*Cyp4v3*^−/−^ Mouse Paper, and the Exon1-*Cyp4v3*^−/−^ Mouse Paper provided any data proving Cyp4v3 protein is expressed in wild type mouse retina [5–7]. Interestingly, the research group which had previously reported CYP4V2 expression in human retina only provided data showing Cyp4v3 protein is expressed in wild type mouse liver in the *Cyp4v3*^−/−^ Mouse Paper [5, 44].

Significantly, immunohistochemical staining reveals that unlike CYP4V2 protein expression in normal human retina, Cyp4v3 protein-specific positive staining is only observed in wild type mouse corneal epithelium, and is not expressed in any part of the retina (Fig. 2). Cross reactivity of the anti-human CYP4V2 antibody to the mouse ortholog Cyp4v3 is confirmed by positive staining in the livers and corneal epithelium of WT mice. Positive staining in other tissues was also observed to confirm antibody cross reactivity (mouse embryo, data not shown). In contrast, human eye stained with the same antibody showed CYP4V2 protein-specific positive staining in the retinal pigment epithelium (RPE); cone outer segments; occasional nuclei in the inner and outer nuclear layers, and the corneal epithelium (Fig. 2). The strongest staining in the human eye was observed in the cone outer segments. The markedly different retinal expression profile between mouse and human retina suggests that unlike CYP4V2 protein in human retina, the murine ortholog Cyp4v3 protein does not play a role in the retina of wild type mice. This indicates that the *Cyp4v3* knockout mouse (whether fed with ND or HFD, and regardless of the knockout strategy) is not an appropriate model for BCD, a human retinal disease resulting from a lack of normal retinal CYP4V2 protein expression.

## Nontransparent science in rare disease research

Publish or perish is a constant pressure on researchers. Publication is a powerful method at scholar’s disposal to demonstrate academic talent to peers [63]. As compared to research on common diseases, rare disease research is not always reviewed with scrutiny, leaving room for nontransparent science. As a patient-turned researcher, I was surprised by the frequency and obviousness of nontransparent science in BCD research. Thus, I felt the need to analyze and discuss various forms of nontransparent science in a direct manner. However, the objective of this article is not to accuse anyone of misconduct but to point out the animal model flaws obscured by nontransparent science and its profound implications for orphan drug development.

Science has no boarders, neither does nontransparent science. As seen in BCD research, nontransparent science can happen irrespective of the researcher’s senority, institutional affiliation or country. Nontransparent science is not equivalent of research misconduct. For example, nontransparent science could be caused by negligence or other reasons without any intention to mislead the readers or to create a false impression. Moreover, it is likely that not all authors of a paper were aware of the nontransparent science in such paper. For example, the authors who generated the knockout mice may be different from the authors who conducted the ophthalmology investigations of the mice and analyzed the data, and vice versa. However, regardless of the form and reason, nontransparent science could affect the validity of the results and conclusions in research publications. Therefore, the rare disease community need to be vigilant about nontransparent science and make independent judgment when reading research publications. The results and conclusions cannot always be taken at face value.

## Preclinical alternatives to animal models

Animals are not the only solution to model human diseases. Advances in cell biology has enabled the development of new human-cell-based alternatives to animal models. Realizing the limitations of animal models The research community, including the NIH, has been driving the development of human cell-based models [25, 27]. In the rare disease field, patient-derived cellular models have been used in preclinical studies to model rare diseases and for the evaluation of potential therapies [64], including retinal diseases [65, 66]. For example, patient-derived induced pluripotent stem (iPS) cell model have been used in preclinical POC study to advance retinal gene therapy for choroideremia to FDA regulated human clinical trial [65]. Patient derived cell model may also play a role in the development of novel gene therapy for treating BCD. For BCD, we went a step further and differentiated patient-derived iPS cells into RPE (iPS-RPE) cells. The iPS-RPE cellular model enables researchers to study BCD directly in the disease target cells of BCD. Other researchers who realized the flaws of the *Cyp4v3*^−/−^ mouse model also used patient iPS-RPE cell lines to study BCD [37, 38]. BCD patients’ iPS-RPE cell lines showed abnormal RPE cell death. This is clinically significant phenotype because BCD is associated with RPE atrophy [40]. Moreover, the use of patient cell model in preclinical POC studies eliminates interspecies risks in clinical translation of preclinical POC results. Recently, the ophthalmology group of the HFD-*Cyp4v3*^−/−^ Mouse Paper published a new paper stating that they are also developing a patient iPS-based cell model for BCD [67].

## A patient’s call for transparent science

My participation in BCD research began two decades ago when I enrolled as a patient in the global study which discovered the *CYP4V2* gene in 2004 [9]. In the ensuing decade, I waited anxiously for a treatment as a patient, but seeing no treatments being developed for this rare disease, I founded Reflection Biotechnologies (ReflectionBio®) in the process of going blind to drive BCD gene and cell therapy research. As a patient-driven biotechnology company, ReflectionBio applies the *By Patients, For Patients*™ approach for rare disease patients to combine efforts and proactively drive medical and scientific research and development to help ourselves and others.

When I began working on BCD gene therapy research as a patient, I knew there would be numerous challenges. One of the biggest challenges I have faced in drug development was to identify a pharmacologically relevant and translatable model of BCD. In light of the *Cyp4v3*^−/−^ Mouse Paper, I spent significant amount of time and efforts on due diligence to determine if this knockout mouse model could be used to develop a BCD gene therapy. Further, during our BCD gene therapy Orphan Drug Designation (ODD) application process, the FDA pointed to the *Cyp4v3*^−/−^ Mouse Paper and asked us to provide results from a preclinical study showing efficacy of our BCD gene therapy in the *Cyp4v3*^−/−^ mouse. Consequently, a considerable amount of time and effort was spent explaining to the FDA that the *Cyp4v3*^−/−^ mouse is not an appropriate model of BCD as this model does not mimic BCD in humans. Although the FDA agreed with our rationale and approved our ODD, these additional efforts resulted in a 6-month delay in the ODD approval. This demonstrated that flawed animal models can misguide medicine regulators and cause delay to drug development.

### Profound implications of nontransparent science for drug development

When nontransparent science obscures animal model flaws, it could have profound implications for orphan drug development and rare disease research, ultimately affecting the patients:

(i) False positive or false negative preclinical POC results. Flawed animal models can generate false positive or false negative preclinical POC study results. Due to interspecies differences, animal models have a poor track record of clinical translatability. Flawed animal model would only further decrease the rate of successful drug development or mistakenly kill drugs that could treat the disease in humans but show no efficacy in animals;
(ii) Blocking drug development based on viable models. Medicine regulators may be misguided by nontransparent science to take flawed animal models as valid models, thereby insisting on using such flawed animal models and not allowing alternative models in POC studies which may provide better clinical translatability. For example, realizing the flaws of the *Cyp4v3*^-/-^ mouse model, researchers developed BCD patient-derived iPS-RPE cell model for BCD. BCD patient iPS-RPE cell model showed abnormal RPE cell death. This is clinically significant phenotype because BCD is associated with RPE atrophy [40]. Moreover, the use of patient cell model eliminates the interspecies risk in clinical translation of POC study results. It would be a shame for the rare disease community if a human cell model developed to overcome the flaws in the mouse model is blocked by nontransparent science; and/or.
(iii) Delay and frustrate orphan drug development. When a drug developer discovers the flaws of an animal model, it could take years to discuss and reach an agreement with the medicine regulator that such animal model is flawed and an alternative model can be used. Delay in drug development could have an impact on the eyes and lives of thousands of patients.

Fully transparent science can not only save limited resources in rare disease research, but also shorten the time and lessen the financial burden of pursuing treatments for these devastating diseases. By candidly and objectively presenting all available data and study limitations, the lessons learned from these studies can have a meaningful impact on rare disease research and the ultimate goal of bringing more hope and treatments to the patients. This article is not to discourage rare disease research, but to urge all researchers to be fully transparent on research and inform on limitations and impact of findings.

### Patients can play a proactive role in driving rare disease research

Today, as a legally blind BCD patient walking with a guide dog, I no longer expect gene therapy would benefit me, let alone my sister who became blind due to this illness 20 years ago. Still, I am using my little remaining vision and personal savings to drive BCD gene therapy, hoping that I can save some fellow patients from the suffering that my sister, myself and countless others have experienced. Our AAV-based BCD gene transfer therapy, recombinant AAV vector encoding human CYP4V2 protein (AAV.CYP4V2), reduced RPE cell death in BCD patients’ iPS-RPE cell lines in preclinical POC study. We are working on several tasks related to investigational new drug application (IND)-enabling work. With personal savings burning out, I am driving BCD gene therapy research inch by inch towards human clinical trials. However, even if we have enough resources to complete the IND-enabling work, we and other BCD drug developers would still be blocked from human clinical trials by the *Cyp4v3*^−/−^ mice and nontransparent science. This time, the regulators will not only point to the *Cyp4v3*^−/−^ Mouse Paper, but also the HFD-*Cyp4v3*^−/−^ Mouse Paper and the Exon1-*Cyp4v3*^−/−^ Mouse Paper and ask the drug developers to use them in preclinical POC studies before we can advance BCD gene therapy to human clinical trial.

Animals cannot talk, but patients can. Perhaps my biggest contribution to BCD research is to be the whistleblower to point out the flaws of various *Cyp4v3*^−/−^ mouse models and the nontransparent science surrounding them. This will pave the way for researchers and drug developers to advance BCD gene therapy or other treatments into human clinical trials without having to be haunted by the mice. Moreover, lessons from BCD research may help prevent nontransparent science from recurring in the research of other rare diseases. There are thousands of rare diseases like BCD for which patients are desperately waiting for a treatment. Transparent science will have a huge impact on their eyes and lives. I hope our genuine intention and efforts in rare disease research will inspire the field to pursue truly transparent science.

Finally, we, the rare diseases patients, can and need to play active roles in rare disease research. Some patients and families have already put their lives in their hands [68]. With faith, relentless efforts and the right partners, WE, the Patients, can play a proactive role in driving research and development to help ourselves and others. As a rare disease patient, I wrote this article with assistive technology. May transparent science benefit the rare disease community.

## Conclusion

Due to interspecies differences, knockout animals do not always mimic human genetic diseases. Nontransparent science in rare disease research could obscure flaws in animal models which lack pharmacological relevance and give rise to the issue of clinical translatability. It can also misguide drug developers and regulators, and delay and frustrate orphan drug development, ultimately affecting patients suffering from these devastating rare diseases.

## Methods and materials

### Imaging of BCD patients’ eyes

#### Spectral domain optical coherence tomography (SD-OCT)

Figure 1 A is an SD-OCT image of a BCD patient (the author) left eye taken using the Heidelberg Spectralis® (Franklin, MA, USA).

#### Fundus photography

Figure 1D is a fundus photography of a BCD patient provided by the BCD patient organization, Invincible Vision, with the patient’s full permission.

#### Other images

Figure 1B C, and related figure legend descriptions are reproduced from the following open access article [14]: Furusato E, Cameron JD, Chan CC. Evolution of Cellular Inclusions in Bietti’s Crystalline Dystrophy. Ophthalmol Eye Dis. 2010;2010(2):9–15.© 2010 the authors, publisher and licensee Libertas Academica Ltd.

### CYP4V2/Cyp4v3 protein ocular expression and localization

#### Mouse and human tissue samples

C57BL/6J wild type (WT) mice were ordered and shipped from the Jackson Laboratory (Sacramento, CA, USA) to Ora, Inc. (Andover, MA, USA). Mice were euthanized by Ora in accordance with accepted American Veterinary Medical Association (AVMA) guidelines at the age of 20 weeks. Eyes and liver of three mice were collected by Ora and shipped to Excalibur Pathology, Inc. (Norman, OK, USA). Human adult eye tissues from cadaveric donors (donor information de-identified) were provided by the Lions Gift of Sight (Saint Paul, MN, USA) or Excalibur Pathology, Inc. from its normal tissue inventory, which was sourced from the Kansas Eye Bank (Wichita, KS, USA).

#### Immunohistochemistry

Immunohistochemistry (IHC) was performed by Paula Keene Pierce, HTL(ASCP)HT of Excalibur Pathology. Mouse eyes and livers and human eyes were processed to paraffin, sectioned at 4–6 μm and placed on slides. Slides were air dried overnight and then placed in a 60 °C oven overnight. Slides were then deparaffinized and brought to water. Antigen retrieval was performed for 20 min with 70% formic acid followed by an additional water rinse and blocking with 2.5% normal horse serum (Vector Labs) for 40 min. Slides were incubated with rabbit anti-human CYP4V2 antibody (Sigma Aldrich #SAB2103886) for 2 h at room temperature or overnight at 4 °C. This antibody has reactivity for human CYP4V2 and mouse Cyp4v3. Following antibody incubation, slides were washed twice with PBS, incubated with Anti-Rabbit Alkaline Phosphatase ImmPress® solution (Vector Labs cat.# MP-5401) for 40 min, washed again with PBS, and stained with Vector® Red (Vector Labs cat. #SK-5100) for 8 to 10 min. After washing with distilled water, the slides were counterstained with hematoxylin, and mounted with coverslips after dehydration. Microscope images of the slides were taken by Excalibur Pathology, Inc. and assessed by Laura Dill Morton, DVM, PhD, DACVP, DABT of Aclairo® Pharmaceutical Development Group, Inc. (Vienna, VA, USA).

## Data Availability

Not applicable.

## Footnotes

1 Reference [45]: This study reported HFD induced a variety of retinal dysfunction in wild-type (WT) mice, including decreased ERGs and decreases in molecules related to cell signaling, calcium homeostasis, and glucose metabolism from retinas in WT-HFD C57BL/6J mice after 12 weeks of HFD feeding started in 5-week-old mice (comparable to 16-week-old mice in the HFD-Cyp4v3^−/−^ Mouse Paper).

2 Reference [46]: This study reported decreased ERGs in WT-HFD C57BL/6J mice after 2-month and 5-month of HFD feeding started in 5-week-old mice, which are comparable to 12-week-old and 24-week-old mice in the HFD-Cyp4v3^−/−^ Mouse Paper, respectively. This paper also suggested WT-HFD mice as an animal model for diabetic retinopathy (DR.

## List of abbreviations

AAV: Adeno-associated virus
AAV.CYP4V2: Recombinant AAV vector encoding human CYP4V2 protein
AD: Alzheimer’s disease
BCD: Bietti’s Crystalline Dystrophy
*Cyp4v3*^−/−^: *Cyp4v3* knockout mouse
DR: Diabetic Retinopathy
ERG: electroretinograms
Exon1- *Cyp4v3*^−/−^: Mouse with exon 1 of the *Cyp4v3* gene knocked out
FDA: the U.S. Food and Drug Administration
FFA: free fatty acids
HFD: high-fat diet
HFD- *Cyp4v3*^−/−^: *Cyp4v3* knockout mouse fed with high-fat diet
ICMJE: the International Committee of Medical Journal Editors
IHC: immunohistochemistry
IND: Investigational new drug application
IPL: inner plexiform layer
iPS: induced pluripotent stem cell
iPS-RPE: RPE cells differentiated from iPS cells
ND: normal diet
NIH: the National Institutes of Health
NIR: near-infrared reflectance imaging
OCT: Optical coherence tomography
ODD: Orphan Drug Designation
OKN: Optokinetic response
POC: Proof-of-concept
RP: Retinitis Pigmentosa
RPE: retinal pigment epithelium
sgRNA: Single guide RNA
WT: wild type
